# Parents’ Challenges beyond the Pediatric Intensive Care Unit: Fraying at the Seams while Balancing between Two Worlds, Home and Hospital

**DOI:** 10.3390/children9020267

**Published:** 2022-02-16

**Authors:** Zainab Alzawad, Frances Marcus Lewis, Amy Walker

**Affiliations:** 1 College of Nursing, The University of Iowa, Iowa City, IA 52242, USA; 2 School of Nursing, University of Washington, Seattle, WA 98195, USA; fmlewis@uw.edu (F.M.L.); walkeraj@uw.edu (A.W.); 3 Public Health Sciences Division, Clinical Sciences Division, Fred Hutchinson Cancer Research Center, Seattle, WA 98109, USA

**Keywords:** PICU, parent, challenges, qualitative, grounded theory

## Abstract

Background: The dominant tradition in studying parents’ responses to their child’s hospitalization in the pediatric intensive care unit (PICU) is to focus on their immediate environment and their children’s well-being. This view of the parents’ experiences fails to describe the broader set of concurrent challenges beyond the PICU that parents carry with them into the PICU. Objectives: This study describes (a) parents’ reactions to juggling their two worlds, home and hospital, when their child is hospitalized in the PICU, and (b) the impact of this juggling on their lives. Methods: Fifteen parents whose child was admitted into a PICU at a tertiary medical center for children in the Pacific Northwest participated in semi-structured interviews. Data analysis and interpretation were guided by grounded theory. Results: The theory grounded in the data and integrated with the core category was Fraying at the Seams while Balancing between Two Worlds, Home and Hospital. Analyses revealed two categories: Bringing My Life to a Halt and Throwing Our Whole Life Off. Conclusion: Even though parents were physically and emotionally present with their child in the PICU, they felt frayed as they concurrently struggled with their physical distance from other children at home. This strain of living in two worlds caused feelings of inadequacy to fulfill their parental role.

## 1. Introduction

Pediatric intensive care (PICU) admissions have increased since the 1980s in the United States, and currently more than 230,000 children are admitted annually to the PICU [1]. The consequences of PICU admission on parents are alarming, including posttraumatic stress disorder (PTSD), anxiety, and depression after PICU discharge [2,3,4]. Around 10% to 42% of parents suffer from PTSD, and 23% to 31% suffer from anxiety after PICU discharge [4].

To date, studies of parental stress in the PICU have predominately focused on identifying sources of parental stress in the PICU while leaving parents’ broader lives beyond the PICU, including parents’ normal daily routines, family, and personal daily life, family roles, and responsibilities, understudied. Qualitative studies have instead been limited to exploring parents’ stress and reactions within the PICU, not beyond it [5,6,7,8,9,10,11,12]. The most commonly reported stressors across the studies were: the overwhelming physical environment [6,8,13], uncertainty about the child’s survivorship and outcomes [5,9,11,13], relationship and communication with staff [6,7,12], and feeling helpless [5,11,13]. According to the systematic review, changes in the parental role in the care of the child were major sources of parental stress [2]. However, the parental role in the care of other children at home while being in the PICU is still understudied.

Little is known about the parents’ challenges outside the PICU and the impact of PICU admission on family dynamics. Three studies reported parents’ challenges outside the PICU as types of PICU stressors [6,9,13]. Cantwell-Bartl and Tibballs [6] conducted a mixed methods study with 29 parents of children with hypoplastic left heart syndrome after their infant’s admission to the PICU. The authors examined parents’ psychological responses in the PICU. Family separation and fathers’ strains in commuting long distances to the hospital were some of the reported parental stressors. The expenses of commuting and financial constraints were also reported in the qualitative interview study by Diaz-Caneja, Gledhill [13], as parents lived in distant areas from the PICU. The authors investigated factors that can influence parents’ psychological outcomes, with 20 parents of children admitted to the hospital (eleven PICU, nine general pediatric units). Hagstrom [9] conducted a mixed methods study with nine parents to describe the sources of stress when their child was admitted to the PICU. Parents were stressed from being separated from their homes and torn between their homes and hospital.

Although the results from these studies add to our understanding about the sources of parental stress in the PICU, they fail to describe the broader set of concurrent challenges beyond the PICU that parents carry with them into the PICU. Therefore, they do not help us to understand parents’ daily life challenges beyond the PICU. The challenges and the impact of critical illness and the PICU experience on parents’ lives are important to investigate so to gain a full understanding of the effect of PICU hospitalization on parents. Shudy, de Almeida [3], in their systematic review, recommended that further studies are needed to identify family disruption, marital cohesion, and financial effects when a child is admitted to the PICU. We argue that identifying parents’ challenges beyond the PICU may be of additional benefit in improving mental health outcomes. Recognition and understanding of the many nuances underpinning parents’ challenges during this stressful period are essential to provide quality interventions to assist parents in managing the impact of the PICU experience on themselves and their families. Identifying parents’ unique challenges when their child is admitted to the PICU is a necessary first step to develop effective and targeted interventions in the future. The aims of this study were to describe (a) parents’ reactions to juggling their two worlds, home and hospital, when their child is hospitalized in the PICU, and (b) the impact of this juggling on their lives.

## 2. Methods

### 2.1. Design

A qualitative grounded theory method was used. Grounded theory methods allowed us to understand the process and context, including events, the circumstances that led to the situation, the action and interaction that parents took to manage their life challenges, and the consequences that resulted from their actions [14]. Using semi-structured face-to-face elicitation interviews, we created a theory of a process grounded in the perspectives of interviewed parents that explained how life challenges affected their lives and family dynamics.

The theory of adjustment to threatening events formed the basis for the core interview questions that were used to generate data in the current study [15]. This theory, however, was not the guiding theory for the analysis. Our goal was to use a grand-level theory to provide a theoretical sensitivity to concepts in the data, identify a connection between concepts, and cast a very wide net to discover and uncover underarticulated social processes [14]. Corbin and Strauss [14] described sensitivity as “having insights as well as being tuned into and being able to pick up on relevant issues, events, and happenings during collection and analysis of the data”.

This study was part of a mixed methods multicomponent study with an overall goal to describe the parents’ experiences in witnessing their child’s hospitalization and environment in the PICU. Understanding both the internal and external processes can provide a comprehensive picture of social processes and parental experiences. The current paper is uniquely focused on the parents’ reported life challenges *outside* the PICU while their child is hospitalized.

### 2.2. Participants and Setting

Participants were a purposive sample of 15 parents who met eligibility criteria whose child was admitted into a PICU with complex medical conditions at a tertiary medical center for children in the Pacific Northwest that serves patients from the largest geographic area in the country (Washington, Alaska, Montana, and Idaho). The medical center also serves as the primary teaching, clinical, and research site in pediatrics in the WA state. The PICU has 32 beds and provides tertiary care for infants, children, and young adults up to age 21 who are critically ill or injured or need complex surgery. Although there is a designated cardiac ICU that takes surgical cardiothoracic pediatric patients, the general PICU takes medical cardiology patients. The general PICU is also able to accommodate critically ill children with complex conditions including but not limited to heart conditions, transplants, major surgeries, cancer and blood disorders, tumors, trauma, neurological, and genetic conditions.

Parents were eligible for inclusion if their child had been admitted for more than 48 h to the PICU. A parent was defined as a person who served in a primary caregiving role and provided sustained care to the child during the child’s stay in the PICU. This included biological parents, foster parents, grandparents, or guardians. It did not include family members or friends providing respite care when the parent was gone. Once a parent’s eligibility was confirmed, the senior author requested permission to recruit the potential study participant from the primary nurse or charge nurse in the PICU. This screening process was required by the Human Subjects Committee at the study center in order to avoid recruiting study participants with a condition unknown to the study author. A total of 21 eligible parents were identified and 15 were enrolled. Six parents were eligible but were not recruited into the study because (a) their child was recently diagnosed with a tumor and the parents were not yet ready to talk about their experience, (b) the child was terminally ill with an unstable condition and was expected to die, or (c) health care providers were in the process of having an end-of-life discussion with the parents. Only one parent of each child was interviewed to gain in-depth and diverse information. Exclusion criteria were children who were readmitted within the last month, received end-of-life treatment, deemed by the clinical team as inappropriate to enroll, and parents with insufficient English to complete questionnaires. Parents of readmitted children were excluded because parents’ experiences with prior admissions could have potentially contaminated their current admission experience. Our intention was to have parents reflect on current challenges, not prior challenges.

### 2.3. Study Procedure

Study approval from the institutional review board of the study center was obtained prior to data collection and all participants were consented. The approved application included management of two issues: methods the investigator would use, if needed, to handle parents’ emotions and distress during the interview. Specifically, if the parent experienced short-lived emotional distress during or after the interview, the protocol stated that the interviewer would empathically respond and invite the parent to reschedule the interview at another time and link the participant to the unit-based social worker. Additionally, the protocol required that if a study participant disclosed suicidal thoughts, the interviewer would disclose that information to the primary nurse. Recruitment occurred in four steps: (a) identifying potential eligible parents through reviewing the electronic medical record of all children admitted to the PICU; (b) confirming parent’s eligibility and obtaining a verbal agreement from the primary or charge nurse prior to approaching each parent; (c) inviting the parent to enroll; (d) obtaining signed informed consent from the parents who agreed to participate in the study.

Semi-structured face-to-face interviews were conducted in the PICU at the child’s bedside or in the “quiet rooms” located in the PICU by the principal author. Interviews were digitally audio-recorded and lasted between 10 and 80 min, with a median of 22 min. The duration of the interviews varied by the extent to which the interviewee elected to elaborate their responses. Two conversation starters were used to begin the interview. Semi-structured core interviews were guided by Taylor’s theory, related to their current PICU challenges. Follow-up questions were used to probe for details and invite parents to elaborate their responses to the five core questions (Table 1). After the interviews, the parents were asked to complete a demographic questionnaire. All interviews were digitally recorded and labeled with participant ID codes. The researchers had no prior relationship with the study participants. The first author conducted the interviews in the PICU. The second author assisted with the analysis and reviewed the quantitative data on the study participants.

### 2.4. Data Analysis

Grounded theory was used to guide data analysis of coding and categorizing data consistent with the recommendation of Corbin and Strauss [14]. Prior to coding, recorded interviews were transcribed verbatim and verified 100% for accuracy against the audio recordings. The first two authors read the transcripts multiple times to gain an in-depth understanding of the content. Coding began with ‘open coding,’ in which the transcribed data were broken down into units and discrete parts. A unit was defined as the complete idea, not a complete sentence. During this line-by-line analysis, the principal investigator’s observation and memos highlighting key points from the interview were written about the identified units. At the same time of open coding, ‘axial coding’ occurred that involved grouping of the fragmented units/codes into subcategories [14]. Then, ‘selective coding’ took place at the same time as the axial coding to develop higher-order or overarching categories [14]. As more data emerged, connections were made between categories and subcategories. Categories were continually examined for similarity and refined to ensure they were mutually exclusive and to reach parsimony in summarizing study data. We identified and refined relationships between categories and linked them. Categories and subcategories were labeled using participants’ words, not words imposed by the authors, and each category was implicitly defined. Analysis began and continued during data collection in order to ground the data through constant comparative analysis. Constant comparative analysis and peer debriefing were carried out at all stages of data coding [14]. After generating the final set of overarching categories and subcategories, along with their definitions, the core category was identified. The core category is the explanatory process that captured the parents’ reported broader life challenges beyond the PICU.

Four strategies protected the trustworthiness of study results: credibility, dependability, confirmability, and transferability [16]. Credibility was protected by conducting face-to-face regular meetings between the first and second author to debrief all aspects of the coding processes, including the units, categories, domains, definitions, and core construct. Meetings were also used to resolve any coding discrepancies and to constantly refine the definitions of the categories. Transcribed interviews were verified by the second author. The second author listened to 50% of the recorded interviews and checked the verbatim transcriptions. Dependability was ensured by maintaining an audit trail, by constant comparative analysis, and by formal peer debriefing during every step of the coding process. The plausibility of study findings and interpretation of study findings involved both authors comparing the interpretation against study categories and domains. Confirmability was also ensured by examining the distinction between categories for their uniqueness and nonoverlapping characteristics, requiring a 100% consensus between the peer debriefer (2nd author) and the primary coder (1st author), and using parents’ words in describing the categories and domains. Parents in this study provided a robust description of their challenges and how they handled them while being in the PICU, which reflected the transferability of the data and results.

## 3. Results

### 3.1. Participants

A total of fifteen parents comprising thirteen mothers and two fathers completed study interviews. Eighty-seven percent of the parents were White and ranged in age from 20 to 60 years (mean 34.40; *SD* = 11.79) with a median age of 34. Most parents were married (67%) and 47% had a college degree. The median length of PICU stay was 4 days, ranging from 2 to 171 days. The length of the PICU stay was the number of days the child was hospitalized in the PICU from admission to the time of interview (i.e., the duration of the PICU admission at the time of interview). Fifty-three percent of the parents had no prior experience with a PICU admission. The vast majority of interviewed parents were not working (73%) at the time of the study. Most parents had additional children at home (73%) whose ages ranged from 5 months to 19 years old.

The PICU-admitted critically ill children (*n* = 15) were mostly male (67%), with a mean age of 40 months (Table 2). PICU admissions were almost evenly split between unplanned (53%) and planned (47%). Some children were admitted to the PICU due to unexpected life-threatening conditions, and some were admitted after a planned or elective surgery.

### 3.2. Core Category

**Figure 1:**
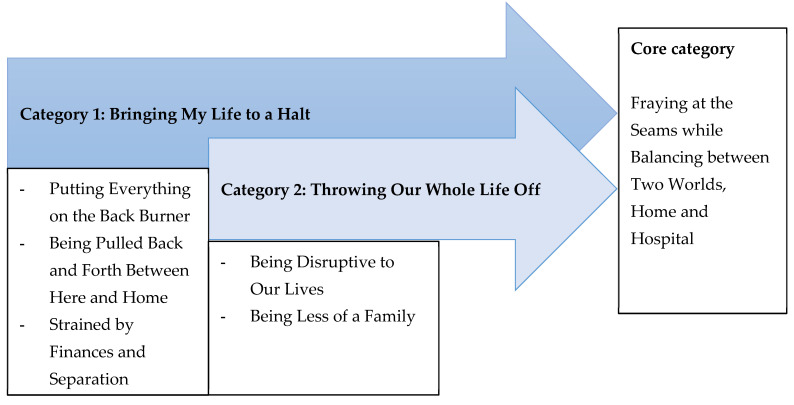
Ground theory of parent’s life challenges outside the PICU.

The core category, Fraying at the Seams while Balancing between Two Worlds, Home and Hospital, summarized parents’ reported challenges. This core category depicted the central phenomenon of the grounded theory. The substantive theory of “balancing between two worlds, home and hospital” represented parents’ efforts to respond to the core problem that family and personal daily life were “thrown off” as the parent’s entire existence focused completely on their hospitalized child’s health and survival. Parents reported their family roles and responsibilities often had to be changed, and normal daily activities and routines had “fallen by the wayside”. Parents were overwhelmed, stressed, and felt like their life was falling apart. Two overarching categories and their subcategories were identified that explained the theoretical process. The process is described in Figure 1. Each step is described below with supporting data.

### 3.3. Category 1: Bringing My Life to a Halt

While parents focused on their sick child, they “put everything else on hold” and stopped “making plans” such as “trips and vacations”. They struggled to have their sick child at the hospital while dealing with their homes and families. Their sick child “took up all of our time and energy and got our attention”. A mother (110) said, “it’s definitely brought like our—my life to a halt. It’s just a new world, is the only way I could think of. It’s a new world with no sleep and high-stress levels, and a good team, but I don’t see my husband as much because he’s working again. During the weekdays, he doesn’t come very long just because he’s gotta go to work, and it can be tiring. So that’s difficult, and, we haven’t really seen any friends or family or anything”. Three subcategories comprised this category.

#### 3.3.1. Putting Everything on the Back Burner

Parents gave less attention to work, other children at home, family, and even self-care because they were fully focused on their sick child in the PICU. Before the PICU admission, parents were working but several had to quit their jobs to be fully present for their child in the PICU. “My child is my full-time job” was a frequent response from mothers. Some mothers explained, “I technically can work, but I’ve chosen not to work so I can be here with him”. Other mothers needed to quit their jobs: “we’ve made the decision for me to leave work and be staying with the boys instead”. Many parents said that their child was their biggest focus or priority over themselves, work, so everything was “put at the back burner”. One mother (130) said, “at least I’ve been really hyper-focused on him, and not really thinking about myself”. Parents also felt that the child got their full attention, and they were always at the PICU making sure that their child is taken care of. One father (106) said: “Being here put everything on hold. Um, work, our other two kids. You know, we don’t get to see them, except on Facetime or, you know, we put everything on hold to focus on getting her better, which we had never been able to do before. It’s always been one or the other, so. It’s kinda nice, actually, that we can”.

Parents felt that they should be constantly present in the PICU for their children. They expressed fear and anxiety to briefly leave their child even for a short time to go for a walk or to the store. Mothers felt “guilty” or “bad getting out or being away from my baby”. A mother (131) said, “I am very leery of leaving my child up in the PICU”.

#### 3.3.2. Being Pulled Back and Forth between Here and Home

Parents reported the challenge of being “pulled back and forth” between the PICU and house, and “balancing” their lives. One father (119) said, “it’s busy, it’s a lotta pull back and forth between what’s going at my house and what’s going here”. Fathers tried to “make sure everybody’s cared for” or they “tried to take care of everybody else’s”. Another father (106) said, “when you multitask you do good at everything, but not great at one”. Many mothers talked about the challenge of “trying to juggle being here and being home, a mom, a mother to my other child, a wife, a homemaker”. They described their houses as being in “shambles” and a lot going on at home when they were in the PICU.

#### 3.3.3. Strained by Finances and Separation

Parents had financial problems or pressure where money caused extreme stress, as they described “financially, things are extremely stressful”. Mothers who talked about their financial difficulties were not working, because they described being in the PICU with their child as a “full-time job”. Parents talked about the difficulty of meeting their basic needs for housing and food. A mother (124) said, “now we can’t afford paying the rent. So we actually are losing our apartment, and me and the baby [other child] will be staying here. His dad lost his job while we were here”. Another mother shared her challenge of renting a “very small house” and was stressed how to “make structural changes to accommodate” her child when he would be discharged home. Parents also struggled with transportation and commuting because they lived distant from the hospital. Other reported financial challenges including debt, bills, gas, and daycare costs for the other children. Parents were also concerned about the challenge when their sick child would be discharged from the PICU and potentially need special equipment such as the electric hospital bed or remodel the home for wheelchair accessibility.

More mothers than fathers reported a lack of support from their partners and families as some fathers were “out of the picture” or their families lived distant from the hospital, which created strains on the relationship and family cohesion. Some mothers said that their child’s father “couldn’t be here” in the PICU, because the father was in the military or lived in a different state, or they were going through a divorce. Another mother (131) said, “my biggest challenge is being away from the rest of my family. So, being far away from our family is very hard”. Mothers felt that they did not “have a lot of families to fall back on” so they did not “have anyone to help”.

### 3.4. Category 2: Throwing Our Whole Life Off

After feeling halted, parents realized that life is a nonstop juggling act. They tried to regain control of their lives. However, they reported that “being in the PICU threw our lives off” out of balance. Admission to the PICU disrupted and scrambled parents’ lives, schedules, physical activities, and family dynamics because of the constant pull back and forth between the PICU and home. Parents reported that they “had to drop off classes,” and “missed going to a conference or work”. Two subcategories comprised this category.

#### 3.4.1. Being Disruptive to Our Lives

Parents reported that PICU was “definitely disruptive to our lives”, including their self-care and physical health such as eating healthy, maintaining their physical activity, sleeping, even their hygiene. As a result, parents reported, “we could feel ourselves fraying at the seams”. One mother had to stop breastfeeding while she was in the PICU. A father (119) said: “Disruptive. Being in the hospital, not just being in the ICU. ICU is more disruptive, but they’re both disruptive to kind of our lives and schedules and that kinda stuff, obviously. And I can’t get a—you know, just being here means I can’t be getting like as much exercise as I’d like. We’re spending a whole lot less time at home, obviously. But we’re taking turns, so we at least get some time home. We’re getting, I mean we’re getting less time with our other son, who is at home with the other parent, so that he’s only getting one parent at any one time. Yeah less time at home and less time at home as a family is the biggest challenge. Even going home by myself, like it’s just—it’s still quiet and kinda sad at home”.

#### 3.4.2. Being Less of a Family

Parents reported that they were trapped in the PICU and socially deprived as they were not able to see their families and friends. A mother (122) said, “I see my husband and the three-year-old kid probably one, maybe two, days a week”. Because parents were mostly at the hospital, parents viewed their home as “kinda sad and very quiet”. They said that being in the PICU affected their family roles and changed their lives at home as a family. They felt isolated and apart from their family and friends. Parents felt “lonely” despite the support from the PICU staff, and that loneliness played into their state of mind. A mother (110) said: “It can be lonely even though there’s so many people in and out, they’re not your friends. I’ve been telling my friends, I’m okay right now but when this is all said and done, I’m gonna need some major therapy”.

As parents were continually present at the PICU with their sick child, they “spent a whole lot less time at home with other kids” and “did not get to see other children except on FaceTime and Skype”. Parents said that PICU admission led them “miss a chunk” of other children’s lives. PICU. One mother (113) said about her five-month-old daughter at home, “the biggest way that it’s affecting my life, and then, um, at first, I wasn’t spending as much time with my other child, so I missed a pretty big chunk of her life. She’s still young, she’s also like at the age where she’s like—she’s starting to sit up and roll over and all of that stuff, and so I’ve missed a good portion of her life. I had to stop breastfeeding while I was here”.

As a result, parents reported some emotional effects on other children at home because they were “only getting one parent at any one time”. The effect of single-parent households confused other children at home as “why my mom isn’t around so much?” and “why my brother isn’t home?” Parents expressed some instability in their household. A mother (124) explained that “spending less time with other children and being home started to wear on everyone at the beginning, everybody kind of rallies, and it’s like, okay, we’ll get through this. But then as it continues to trudge on, people start to get more tired”.

Parents tried to “balance between child and husband and home and here”. They attempted to compensate for their parental absence by “spending lots of time with the kiddo while being at home,” and “making sure both kids get equal time, which is not really happening and hard to deal with”. However, parents felt “guilty” and inadequate in their parental role towards other children at home. A mother (124) explained, “I’m working on forgiving myself for being a position where I can’t be a full-time mom, I feel like, to both of my boys, and feel kind of like a part-time mom to two boys, so half my time here and half my time there. So trying to forgive myself for that”.

## 4. Discussion

Parents faced a cascade of challenges that went far beyond the walls of the PICU. Their child’s hospitalization impacted parents’ family and home life, putting parents’ lives into turmoil. As parents were constantly present in the PICU, they were physically and emotionally distant from their other children at home, partners, friends, and family members. Consistent with findings described by Hagstrom [9], parents felt that they were “being split” and “torn between being home and being at the hospital”. Hagstrom [9] also reported that “the constant pull” between wanting to be home with other children and in the PICU with the ill child was the most stressful thing for parents. Parents talked about feeling reluctant to leave the hospital despite knowing that their child was receiving great care. Nevertheless, the prior study showed that continuously staying in the PICU can be emotionally and physically draining for parents [5]. Abela et al. [2] argued that although PICU guidelines foster family-centered care (FCC) and the presence of parents to be with their child in the PICU, the clinical team needs to consider that parents will be potentially exposed to distressing and traumatizing situations in the PICU.

Parents in the current study were “frayed at the seams” despite their attempts to balance their time in the PICU with the other children, family, and work. Parents said they could look like they were handling the PICU admission well, but if we just looked a little closer, we would see that damage was being done that threatened the fabric of their life. “Being pulled back and forth between the PICU and home” created two worlds in which parents were forced to live. For the most part, parents tried to find strategies to compensate for the gaps between these two worlds, attempting to restore their lives by spending equal or more time at home and using virtual communication with other children at home. Evidence showed that siblings of critically ill children may experience stress, confusion, loneliness, jealousy, sadness, and physical and behavioral changes related to their parental absences [17]. In the current study, parents reported that their children at home were emotionally affected and confused.

## 5. Study Limitations

Study results should be interpreted with caution. Non-English-speaking parents were excluded; results may not represent the experiences of non-English-speaking participants or participants from diverse ethnic or racial backgrounds. Second, the sample of parents was almost exclusively mothers; experiences and challenges of fathers may be different from those of mothers. Third, the one-time interview is limited in its ability to capture parents’ challenges over time in the PICU. The interview questions focused on both the parents’ immediate situation and their entire experience in the PICU beginning with the child’s point of admission. Future research needs to consider framing the parents’ questions to specific time frames in order to allow for a systematic comparison over time. Future research should also purposively sample parents of children recently admitted as well as those who had been admitted into the PICU for a longer period of time. Moreover, future studies should consider a longitudinal design in order to capture changes over time in the parents’ responses. The cross-sectional design of the current study precludes such a comparison. Finally, future research needs to examine supportive intervention to alleviate parents’ distress while fostering FCC. Evidence from a prior study showed that listening to mothers of preterm infants significantly reduced their depressive and anxiety symptoms [18]. Attending to parents’ stories may help parents express their feelings, which may ultimately contribute to self-soothing, self-reflection, and tension regulation [19,20].

## 6. Conclusions

Parents’ lives were brought to a halt and their lives were thrown off. Parents were shattered by being pulled apart between the hospital, home, and work. Even though parents were physically and emotionally present with their ill child in the PICU, they felt frayed and did not know how to help their ill child even as they concurrently struggled with their physical distance from other children at home. This strain of living in two worlds caused feelings of inadequacy and incompetence to fulfill their parental roles.

## Figures and Tables

**Table 1:** Interview questions.

Conversation Starter	Core Interview Questions	Follow-Up Probes
- What happened that brought your child to the intensive care unit? - What words capture for you what it means to have your child admitted to the PICU?	- What are the biggest challenges you are facing right now? - What challenges, if any, are you not able to manage right now? - How are you dealing with the biggest challenges? - What else is going on in your life while your child is in the PICU? - How is your child’s admission to the PICU affecting your life right now?	- Can you tell me more about this situation/event/challenge/experience? - What do you mean with …? - How this challenge/situation affected you personally? - How this challenge/situation affected your life at home/work? - What made this situation/experience/challenge more difficult?

**Table 2:** Demographic characteristics of the children (*n* = 15).

ID.	Child Age	Gender	Diagnoses	LOS (Days)	Previous PICU
102	13 months	Male	Bone marrow transplant, acute respiratory failure	60	Yes
103	2 months	Male	Acute bronchiolitis, E. coli UTI	4	No
106	8 years	Female	Acute respiratory failure	4	Yes
108	8 years	Female	Respiratory distress, developmental disorder	3	No
110	11 months	Male	Alagille syndrome, liver transplant	11	No
113	39 months	Male	Acute respiratory failure	81	No
114	4 months	Female	Chronic GERD	9	No
117	9 months	Male	Acute respiratory failure, chronic illness	4	Yes
119	35 months	Male	Wilms tumor	4	Yes
122	16 years	Male	Kidney transplant	4	No
124	6 months	Male	Chronic respiratory failure, chronic illness	171	Yes
127	1 month	Female	Respiratory distress, chronic illness	2	No
129	13 months	Male	Tracheomalacia, chronic illness	8	Yes
130	5 years	Male	Brain tumor	2	No
131	17 years	Female	Brain tumor	14	No

## Data Availability

The data are not publicly available due to privacy and ethical restrictions. The data presented in this study are available on request from the corresponding author.

## References

[1] Watson R.S., Hartman M.E., Wheeler D.S., Wong H.R., Shanley T.P. (2014). Epidemiology of Critical Illness. Pediatric Critical Care Medicine.

[2] Abela K.M., Wardell D., Rozmus C., LoBiondo-Wood G. (2020). Impact of pediatric critical illness and injury on families: An updated systematic review. J. Pediatr. Nurs..

[3] Shudy M., de Almeida M.L., Ly S., Landon C., Groft S., Jenkins T.L., Nicholson C.E. (2006). Impact of pediatric critical illness and injury on families: A systematic literature review. Pediatrics.

[4] Yagiela L.M., Lauren M., Carlton E.F., Mert K.L., Odetola F.O., Cousiono M.K. (2019). Parent Medical traumatic stress and associated family outcomes after pediatric critical illness: A systematic review. Pediatr. Crit. Care Med..

[5] Alzawad Z., Lewis F.M., Kantrowitz-Gordon I., Howells A.J. (2020). A qualitative study of parents’ experiences in the pediatric intensive care unit: Riding a roller coaster. J. Pediatr. Nurs..

[6] Cantwell-Bartl A.M., Tibballs J. (2013). Psychosocial experiences of parents of infants with hypoplastic left heart syndrome in the PICU. Pediatr. Crit. Care Med..

[7] Colville G., Darkins J., Hesketh J., Bennett V., Alcock J., Noyes J. (2009). The impact on parents of a child’s admission to intensive care: Integration of qualitative findings from a cross-sectional study. Intensive Crit. Care Nurs..

[8] Dahav P., Sjöström-Strand A. (2018). Parents’ experiences of their child being admitted to a paediatric intensive care unit: A qualitative study-like being in another world. Scand. J. Caring Sci..

[9] Hagstrom S. (2017). Family stress in pediatric critical care. J. Pediatr. Nurs..

[10] Oxley R. (2015). Parents’ experiences of their child’s admission to paediatric intensive care. Nurs. Child Young People.

[11] Stratton K.M. (2004). Parents experiences of their child’s care during hospitalization. J. Cult. Divers..

[12] Latour J.M., van Goudoever J.B., Schuurman B.E., Albers M.J.I.J., van Dam N.A.M., Dullaart E., van Heerde M., Verlaat C.W.M., van Vught E.M., Hazelzet J.A. (2011). A qualitative study exploring the experiences of parents of children admitted to seven Dutch pediatric intensive care units. Intensive Care Med..

[13] Diaz-Caneja A., Gledhill J., Weaver T., Nadel S., Garralda E. (2005). A child’s admission to hospital: A qualitative study examining the experiences of parents. Intensive Care Med..

[14] Corbin J., Strauss A.L. (2015). Basics of Qualitative Research: Techniques and Procedures for Developing Grounded Theory.

[15] Taylor S.E. (1983). Adjustment to threatening events: A theory of cognitive adaptation. Am. Psychol..

[16] Polit D.F., Beck C.T. (2012). Resource Manual for Nursing Research: Generating and Assessing Evidence for Nursing Practice.

[17] Rozdilsky J.R. (2005). Enhancing sibling presence in pediatric ICU. Crit. Care Nurs. Clin. N. Am..

[18] Segre L.S., Siewert R.C., Brock R.L., O’hara M.W. (2013). Emotional distress in mothers of preterm hospitalized infants: A feasibility trial of nurse-delivered treatment. J. Perinatol..

[19] Frattaroli J. (2006). Experimental disclosure and its moderators: A meta-analysis. Psychol. Bull..

[20] Kircanski K., Lieberman M.D., Craske M.G. (2012). Feelings into words: Contributions of language to exposure therapy. Psychol. Sci..

